# Risk factors, clinical presentations and predictors of stroke among adult patients admitted to stroke unit of Jimma university medical center, south west Ethiopia: prospective observational study

**DOI:** 10.1186/s12883-019-1409-0

**Published:** 2019-08-07

**Authors:** Ginenus Fekadu, Legese Chelkeba, Ayantu Kebede

**Affiliations:** 1 Department of Pharmacy, Institute of Health Sciences, Wollega University, P. O Box 395, Nekemte, Ethiopia; 2 School of Pharmacy, Institute of Health, Jimma University, Jimma, Ethiopia; 3 Department of Epidemiology, Institute of Health, Jimma University, Jimma, Ethiopia

**Keywords:** Stroke, Risk factors, Clinical presentation, Predictors, Jimma, Ethiopia

## Abstract

**Background:**

Stroke is the second-leading global cause of death behind heart disease in 2013 and is a major cause of permanent disability. The burden of stroke in terms of mortality, morbidity and disability is increasing across the world. It is currently observed to be one of the commonest reasons of admission in many health care setups and becoming an alarming serious public health problem in our country Ethiopia. Despite the high burden of strokes globally, there is insufficient information on the current clinical profile of stroke in low and middle income countries (LMICs) including Ethiopia. So, this study was aimed to assess risk factors, clinical presentations and predictors of stroke subtypes among adult patients admitted to stroke unit of Jimma university medical center (JUMC).

**Methods:**

Prospective observational study design was carried out at stroke unit (SU) of JUMC for 4 consecutive months from March 10–July 10, 2017. A standardized data extraction checklist and patient interview was used to collect data. Data was entered into Epi data version 3.1 and analyzed using SPSS version 20. Multivariable logistic regression was used to identify the predictors of stroke subtypes.

**Result:**

A total of 116 eligible stroke patients were recruited during the study period. The mean age of the patients was 55.1 ± 14.0 years and males comprised 62.9%. According to world health organization (WHO) criteria of stroke diagnosis, 51.7% of patients had ischemic while 48.3% had hemorrhagic stroke. The most common risk factor identified was hypertension (75.9%) followed by family history (33.6%), alcohol intake (22.4%), smoking (17.2%) and heart failure (17.2%). The most common clinical presentation was headache complained by 75.0% of the patients followed by aphasia 60.3% and hemiparesis 53.4%. Atrial fibrillation was the independent predictor of hemorrhagic stroke (AOR: 0.08, 95% CI: 0.01–0.68).

**Conclusion:**

The clinical characteristics of stroke in this set up were similar to other low- and middle-resource countries. As stroke is a high priority chronic disease, large-scale public health campaign should be launched focusing on public education regarding stroke risk factors and necessary interventions.

**Electronic supplementary material:**

The online version of this article (10.1186/s12883-019-1409-0) contains supplementary material, which is available to authorized users.

## Background

Stroke is acute clinical event of focal or global neurological disturbance related to impairment of cerebral circulation, which lasts longer than 24 h resulting in death with no known cause other than vascular origin. Without blood to supply oxygen and to remove waste products, brain cells quickly begin to die [1–4]. Stroke is the second-leading global cause of death behind heart disease in 2013 and is a major cause of permanent disability [5–7]. Currently, the burden of stroke in terms of mortality, morbidity and disability is increasing across the world [8, 9]. Additionally, data from Global Burden of Diseases, Injuries, and Risk Factors Study (GBD) of 2010 revealed that stroke is the leading cardiovascular disease (CVD) which causes mortality and disability in sub-Saharan Africa (SSA) and other low and middle income countries (LMICs) [10].

Risk factors for stroke can be classified as modifiable and non-modifiable. Age, sex, family history and race/ethnicity are non-modifiable risk factors; while hypertension, smoking, diet, and physical inactivity are among some of identified modifiable risk factors [11]. Different risk factors apply to an African population in the development of stroke [12]. Africa might be increasingly affected by high burden of stroke and other vascular diseases due to health transitions in line with ever-changing social, economic and demographic patterns [13]. Additionally, the poor are increasingly affected by stroke, which can be attributable to the changing population exposures to risk factors and inability to afford the high cost of stroke care [14]. Yet, only little data about context-specific risk factors for prioritizing interventions to reduce the stroke burden in sub-Saharan Africa is available [15, 16].

Compared to developed countries, the percentage of hemorrhagic stroke (HS) mortality rate was higher in SSA and other LMICs [10, 17, 18]. There have been variations in the prevalence of major risk factors among the stroke subtypes, demonstrating that knowledge of pathophysiology is crucial for the right management and care of the patients [19]. In addition to highest burden of stroke risk factors in LMICs, the racial or genetic factors also plays key roles in the pathogenesis of stroke. For example, hypertension and diabetes mellitus (DM) appear to be more prevalent among black races as compared to white races [17]. Currently even though several modifiable risk factors are becoming significant, hypertension is still the most common risk factor globally including our country [20].

Stroke is currently observed to be one of the commonest reasons of admission in many health care setups and becoming an alarming serious public health problem in our country Ethiopia [21, 22]. Under-diagnosing of hypertension and other risk factors, delayed presentation to the hospital, poor risk factors control and failure to adhere to the treatments are some of the major challenges that needs to be addressed [21, 23]. Etiologic investigation for stroke was infrequently performed due to lack of systematic cardiological examinations and brain imaging, most of the time for economic reasons and unavailability of the instruments [24]. The findings of the studies done in Ethiopia frequently changed from one another with respect to various demographic profiles, location and risk factors [21]. Most of the data’s regarding stroke that used in the management, follow-up and prevention of stroke come from studies in developed countries [22]. Thus, in our country we haven’t pooled data on prevalence, risk factors and outcome of the stroke.

The shortage of data specific to the Ethiopian setting limits the formulation of well-designed response and management of stroke [21]. So it is imperative that a lot has to be done to overcome the current challenges concerning the risk factors and clinical profile of stroke in Ethiopia [22]. Hence this study will generate evidences for improving the prevention strategy of stroke and guide health authorities to halt or reduce the devastating effects of stoke at different sectors of our community by having overview knowledge of clinical characteristics of stroke. This study data was part of huge study project done in stroke unit (SU) of Jimma university medical center (JUMC) with novel and extensive findings focusing on stroke. Hence, this study was aimed to assess risk factors, clinical presentation and predictors of stroke subtypes among adult patients admitted to SU of JUMC.

## Methods

Since this data was part of study previously described by Fekadu etal [24], we have used similar methods. Additionally, the study participants in this finding share similarity with previously published articles of the same study project. Prospective observational study design was conducted at SU of JUMC located at south-west Ethiopia for 4 consecutive months from March 10–July 10, 2017. All adult patients (> 18 years) diagnosed to had stroke clinically or by brain imaging and admitted to SU of JUMC during the study period were included. Those not willing to give an informed consent, died before evaluation, changed diagnosis of stroke, transformed stroke and with hematomas were excluded [23, 24].

### Data collection tool and procedure

Data collection was carried out by two trained nurses and one internal medicine resident. Data collectors collect data using interviewer administered questionnaire and standardized data extraction form from the case records of the patients. Data collection tool (Additional file 1) was developed based on the previous study findings done at different sites and using the WHO step wise approach to stroke surveillance [25]. The necessary history used for the study was taken from the patient and/or caregivers by the language they understood. To ensure quality of data, the data abstraction tool was developed in English, translated to local language (Amharic and Afan Oromo) and back translated into English to check its consistency. The data collection form was used to collect data on the sociodemographic characteristics, clinical characteristics of patients such as risk factors, clinical presentation and subtypes of stroke.

### Data processing and analysis

The data was entered to Epidata version 3.1 and analyzed using statistical package for the social sciences (SPSS) version 20. Descriptive statistics such as proportions, means, standard deviations, medians and Interquartile ranges were calculated to describe the independent variables. During candidate selection because of adequate significant variables were obtained at *P* < 0.05, it was considered as cut off point for candidate selection for multivariable logistic regression analysis model with backward stepwise approach to identify the independent predictors of stroke subtypes. The data was summarized using odds ratio (OR) and 95% confidence interval. Confidence interval which doesn’t contain 1 and predictor variables with *p* value less than 0.05 was considered statistically significant.

### Operational definition

Alcohol abuse/ consumption: on average ≥ 2 drinks/day for males and ≥ 1 drinks for females (previous drinker: ex drinker for more than 1 year) [26].

Diabetes mellitus: If the patient was previously on oral hypoglycemic agents/insulin treatment or had the diagnosis of any type of DM or FBS ≥ 126 mg/dl or had a documented RBS ≥ 200 mg/dl or glycosylated hemoglobin of ≥6.5% [7, 27–29]

Dyslipidemia or hyperlipidemia: Previous had history of hyperlipidemia or using lipid lowering medication or total cholesterol ≥200 mg/dl, LDL cholesterol ≥100 mg/dl, and HDL-cholesterol < 40 mg/dl for men or < 50 mg/dl for women, and/or serum triglyceride level ≥ 150 mg/dl [27, 30].

Hypertension: Previously receiving antihypertensive medication or when the patient was previously diagnosed with hypertension or detecting blood pressure of ≥ 140/90 mm/Hg for two measurements [7, 27–29].

Obesity: According to the WHO, Body Mass Index (BMI) ≥ 30 kg/m ^2^ [28].

- Central obesity: Waist circumference greater than 102 cm in men and 88 cm in women [28].

Smoker: On average 2 cigarettes per day in men and 1 per day in women

- Former smoker: who abstained from smoking for greater than 1 years [31].
- Current smoker: smoking within 1 year ago [31].

## Result

One hundred twenty five patients were admitted to SU of JUMC with suspected diagnosis of stroke and 9 patients were excluded from the study during the study period. From 116 study participants included in the study; history was obtained solely from 11 patients (9.5%), from the patient and caregiver in 50 cases (43.1%), and solely from caregivers in 55 cases (47.4%). According to WHO criteria 51.7% patients had ischemic type of stroke (IS) while 48.3% had hemorrhagic stroke (HS). Of the total 116 patients, 61 patients evaluated with CT scan of the brain and the rest 55 patients were evaluated clinically to have stroke [24].

### Patient characteristics

The mean age of the patients was 55.1 ± 14.0 years and 65 (56.0%) were in age of group of 45–65 years. Males comprised of 73 (62.9%) with male: female ratio of 1.70:1. Majority of the participants (42.2%) had informal education and 85.3% of patients were independent at home during pre-stroke. Majority of the patients had normal mean body mass index (BMI) (63.8%) and 15.5% of the patients were overweight [23]. Regarding the food habit of the patients during the pre-stroke, 81.9% were mixed diet users **(**Table 1**).**

**Table 1:** Patient characteristics among adult stroke patients admitted to stroke unit of JUMC from March 10–July 10, 2017

Patient characteristics		Total patients (*n* = 116)	Ischemic stroke (*n* = 60)	Hemorrhagic stroke (*n* = 56)
*Age* (*years*)	< *45*	26 (22.4%)	10 (16.7%)	16 (28.6%)
	*45*–*65*	65 (56.0%)	34 (56.7%)	31 (55.4%)
	> *65*	25 (21.6%)	16 (26.7%)	9 (16.1%)
*Sex*	*Male*	73 (62.9%)	38 (63.3%	35 (62.5%
	*Female*	43 (37.1%)	22 (36.7%)	21 (37.5%)
*Residence*	*Rural*	84 (72.4%)	43 (71.7%)	41 (73.2%)
	*Urban*	32 (27.6%	17 (28.3%)	15 (26.8%)
*Marital status*	*Married*	104 (89.7%)	54 (90.0%)	50 (89.3%)
	*Widow*	11 (9.5%)	5 (8.3%)	6 (10.7%)
	*Divorced*	1 (0.9%)	1 (1.7%)	0 (0%)
*Religion*	*Muslim*	71 (61.2%)	40 (66.7%)	31 (55.4%
	*Orthodox*	35 (30.2%)	15 (25.0%)	20 (35.7%)
	*Protestant*	9 (7.8%)	4 (6.7%)	5 (8.9%)
	*Traditional belief*	1 (0.9%)	1 (1.7%)	0 (0%)
*Education status*	*Unable to read and write*	42 (36.2%)	20 (33.3%)	22 (39.3%)
	*Able to read and write, informal education*	49 (42.2%)	28 (46.7%)	21 (37.5%)
	*Elementary school* (*1–8*)	17 (14.7%)	10 (16.7%)	7 (12.5%)
	*Secondary school* (*9–12*)	3 (2.6%)	1 (1.7%)	2 (3.6%)
	*College/university or above*	5 (4.3%)	1 (1.7%)	4 (7.1%)
*Occupational status* (*over the last 1 year*)	*Agriculture / farmer*	44 (37.9%)	27 (45.0%)	17 (30.4%)
	*Homemaker/ housewives*	41 (35.3%	20 (33.3%)	21 (37.5%)
	*Merchant*	11 (9.5%)	7 (11.7)	4 (7.1%)
	*Retired*	6 (5.2%	2 (3.3%)	4 (7.1%)
	*Government employee*	5 (4.3%)	1 (1.7%)	4 (7.1%)
	*Other own business work*	5 (4.3%)	2 (3.3%)	3 (5.4%)
	*Skilled/unskilled manual labor/ daily worker*	4 (3.4%)	1 (1.7%)	3 (5.4%)
*Body mass index* (*BMI*)(*kg/m*^*2*^)	*Mean* ± *standard deviation*	21.2 ± 3.4	21.4 ± 3.7	21.1 ± 3.0
	≤ *18.5* (*underweight*)	24 (20.7%	13 (21.7%)	11 (19.6%)
	*18.6–24.9* (*normal*)	74 (63.8%)	34 (56.7%)	40 (71.4%)
	*25.0–29.9* (*overweight*)	18 (15.5%)	13 (21.7%)	5 (8.9%)
*Living situation during pre-stroke*	*Independent at home*	99 (85.3%)	47 (78.3%)	52 (92.9%)
	*Dependent at home*	14 (12.1%)	10 (16.7%)	4 (7.1%)
	*hospital/ health center*	3 (2.6%)	3 (5.0%)	0 (0%)
*Feeding habits*	*Mixed diet*	95 (81.9%)	47 (78.3%)	48 (85.7%)
	*Non vegetarian*	15 (12.9%)	10 (16.7%)	5 (8.9%)
	*Vegetarian*	6 (5.2%)	3 (5.0%	3 (5.4%)

### Risk factors for stroke subtypes

Risk factors were identified in 114 (98.3%) patients; 59 (98.3%) of IS and 55 (98.2%) of HS patients. The most common risk factor identified was hypertension in 88 (75.9%) patients followed by family history in 39 (33.6%), alcohol intake 26 (22.4%) and smoking 20 (17.2%). Thirty six patients (83.7%) of IS and 34 (75.6%) of HS patients had a pre-stroke knowledge of being hypertensive. Twenty eight patients (24.1%) had no current and previous history of hypertension [17 (28.3%) of IS and 11(19.6%) of HS patients] **(**Table 2**).**

**Table 2:** Risk factors of stroke subtypes among adult patients admitted to stroke unit of JUMC from March10–July 10, 2017

Stroke risk factors		Total patients (*n* = 114)	Ischemic stroke (*n* = 59)	Hemorrhagic stroke (*n* = 55)	OR (*P* value)
*Hypertension*	*New diagnosis*	18 (20.5%)	7 (16.3%)	11 (24.4%)	1.00
	*Previous history*	70 (79.5%)	36 (83.7%)	34 (75.6%)	0.345
*Family history (n = 39)*	*Hypertension*	21 (53.8%	12 (60.0%)	9 (47.4%)	0.584
	*Sudden death*	15 (38.5%	7 (35.0%)	8 (42.1%)	0.675
	*Diabetes mellitus*	10 (25.6%)	6 (30%)	4 (21.1%)	0.585
	*Ischemic heart disease*	5 (12.8%)	2 (10%)	3 (15.8%)	0.595
	*Stroke*	2 (5.1%)	2 (10%)	0 (0%)	0.999
*Alcohol intake*	*Former drinker*	22 (84.6%	10 (100%)	12 (75.0%)	1.00
	*Current alcohol use*	4 (15.4%)	0 (0%)	4 (25.0%)	0.999
*Smoking*	*Current smoker*	10 (50.0%)	1 (16.7%)	9 (64.3%)	0.074
	*Former smoker*	10 (50.0%)	5 (83.3%	5 (35.7%)	1.00
*Heart failure*		20 (17.2%)	16 (26.7%)	4 (7.1%)	0.009
*Atrial fibrillation*		19 (16.4%)	15 (25.0%)	4 (7.1%)	0.014
*Overweight/central obesity*		18 (15.5%)	13 (21.7%)	5 (8.9%)	0.066
*Hematological disorders (n = 17)*	*Polycythemia*	7 (41.2%)	5 (45.5%)	2 (33.3%)	0.407
	*Anemia*	6 (35.3%)	4 (36.4%)	2 (33.3%)	0.459
	*Thrombocytopenia*	5 (29.4%)	3 (27.3%)	2 (33.3%)	0.706
	*Thrombocytosis*	1 (5.9%)	1 (9.1%)	0 (0%)	–
*Diet (low fruit and vegetable)*		15 (12.9%)	10 (16.7%)	5 (8.9%)	0.221
*Coronary disease (CHD, IHD)*		14 (12.1%)	11 (18.3%)	3 (5.4%)	0.043
*Hypertensive heart disease (HHD)*		13 (11.2%)	10 (16.7%)	3 (5.4%)	0.066
*Physical inactivity/sedentary life*		13 (11.2%)	9 (15.0%)	4 (7.1%)	0.189
*Migraine or headache*		12 (10.3%)	2 (3.3%)	10 (17.9%)	0.021
*Psychosocial stress*		10 (8.6%)	2 (3.3%)	8 (14.3%)	0.053
*Traumatic brain /head injury*		9 (7.8%)	2 (3.3%)	7 (12.5%)	0.085
*Previous stroke*		9 (7.8%)	8 (13.3%)	1 (1.9%)	0.048
*Diabetes mellitus*	*Newly diagnosed*	4 (50%	1 (25%)	3 (75%)	1.00
	*Previous history*	4 (50%)	3 (75%)	1 (25%)	0.178
*Contraceptive drug use*		8 (6.9%)	5 (8.3%)	3 (5.4%)	0.530
*Hyperlipidemia (all newly diagnosed)*		6 (5.2%)	5 (8.3%)	1 (1.8%)	0.148
*Infective meningitis (TB / bacterial meningitis)*		5 (4.3%)	1 (1.7%)	4 (7.1%)	0.182
*Oral anticoagulant*		5 (4.3%)	5 (8.3%)	0 (0%)	0.999
*Nephrotic syndrome (Chronic kidney diseases)*		4 (3.4%)	3 (5.0%)	1 (1.8%)	0.364
*Epilepsy/seizure*		3 (2.6%)	2 (3.3%)	1 (1.8%)	0.527
*Other Malignancy/ history of other cancer*		3 (2.6%)	1 (1.7%)	2 (3.6%)	0.528
*Preeclampsia-eclampsia*		3 (2.6%)	2 (3.3%)	1 (1.8%)	0.605
*Other cardiovascular diseases such as DCM*		3 (2.6%	3 (5.0%)	0 (0%)	0.999
*VTE (hypercoagulable state)*		2 (1.7%)	1 (1.7%)	1 (1.8%)	0.961
*Hyperuricemia*		1 (0.9%)	1 (1.7%)	0 (0%)	–
*Thyrotoxicosis*		1 (0.9%)	1 (1.7%)	0 (0%)	–
*Chronic obstructive pulmonary disease (COPD)*		1 (0.9%)	1 (1.7%)	0 (0%)	–
*Valvular heart disease*		1 (0.9%)	1 (1.7%)	0 (0%)	–
*HIV*		1 (0.9%)	1 (1.7%	0 (0%)	–
*Other comorbidities*		3 (2.6%)	3 (5%)	0 (0%)	0.999

*CHD* Coronary Heart Disease, *DCM* Dilated Cardiomyopathy, *HIV* Human Immune Virus, *IHD* Ischemic Heart Disease, *OR* Odds ratio, *TB* Tuberculosis, *VTE* Venous thromboembolism

About 18 (20.5%) of the patients had no prior knowledge of being hypertensive, but diagnosed in hospital during admission for stroke. From 46 patients with no previous history of hypertension including newly diagnosed, 19 (41.3%) were never had their blood pressure measured and the remaining measured but was in normal range.

Among the patients with recorded history of hypertension, the median duration of hypertension prior to stroke diagnosis was 3 years (ranged 0.04 to 25 years). Of 70 patients with pre-existing hypertension, 27 (38.6%) were on anti-hypertensive medications, 24 (34.3%) of the patients were discontinued their antihypertensive medication and 19 (27.1%) hadn’t started antihypertensive medication before stroke occurrence. From 51 patients previously started antihypertensive medication the median duration since the medication started was 3 years. From the 27 patients that were on antihypertensive medication during hospital arrival, 19 (70.4%) of the patients’ blood pressure was not controlled. The median month since discontinuation of their antihypertensive medications before onset of stroke was 2.5 months (ranged 0.5 to 48 months).

Diabetes Mellitus was identified as co-morbidity in 8 patients (4 of them previously diagnosed). It was more prevalent in males and in middle age group, but there was no statistically significant difference between stroke subtypes (*p* = 0.178). Among the patients with previous history of diabetes, the mean duration of diabetes prior to stroke was 5.3 years (ranged 3 to 9 years). Although all previously diagnosed patients were on anti-diabetics, only 1 patient’s blood glucose was controlled (RBS ≤ 200 mg/dl) during hospital arrival.

Physical inactivity/ sedentary life was detected in 13 (11.2%) patients, the remaining patients had habit of physical activity. Of those who had physical activity, 101 (98.1%) had work related aerobic physical activity and 2 (1.9%) had aerobic/planned physical activity. From nine patients (7.8%) who gave a history of previous stroke, eight of them were ischemic stroke patients. From those patients who had previous history of stroke, one patient had history of hypertension for 25 years.

Alcohol consumption and cigarette smoking was less prevalent in IS than HS patients, which was statistically significant among smokers (*p* = 0.038). Majority of the patients who used alcohol were former drinker before 1 year (84.6%), but no difference in smoking status of the patients between the current and previous smokers. Seventy stoke patients (83.6%) had two or more risk factors for stroke, while 17 (14.7%) had one identified risk factor. In addition 17 (14.7%) patients had more than five identified risk factors. With this, the average risk factor for the patient was 3.38 (ranged 0 to 9) risk factors.

### C**linical presentation of stroke patients**

The most common clinical presentation was headache complained by 87 (75.0%) patients followed by aphasia 70 (60.3%) and hemiparesis 62 (53.4%). Most of ischemic stroke patients presented with headache (71.7%), aphasia (60.0%) and facial palsy (58.3%). Similarly, the common clinical presentations among hemorrhagic stroke patients was headache (78.6%) followed by aphasia (60.7%) and vomiting (57.1%) **(**Table 3).

**Table 3:** Clinical presentations of stroke subtypes among adult patients admitted to stroke unit of JUMC from March 10–July 10, 2017

Clinical presentations	Total patients (*N* = 116)	Ischemic stroke (*n* = 60)	Hemorrhagic stroke (*n* = 56)	OR (*P* value)
*Headache*	87 (75.0%)	43 (71.7%)	44 (78.6%)	0.392
*Aphasia/ dysphasia*	70 (60.3%)	36 (60.0%)	34 (60.7%)	0.937
*Hemiparesis*	62 (53.4%)	31 (51.7%)	31 (55.4%)	0.691
*Facial palsy*	60 (51.7%)	35 (58.3%	25 (44.6%)	0.142
*Vomiting*	54 (46.6%)	22 (36.7%)	32 (57.1%)	0.028
*Bladder/ urinary incontinence*	44 (37.9%)	21 (35.0%)	23 (41.1%)	0.501
*Decreased level of consciousness*	43 (37.1%	21 (35.0%)	22 (39.3%)	0.633
*Hemiplegia*	38 (32.8%)	31 (51.7%)	31 (55.4%)	0.589
*Swallowing difficulty / dysphagia*	26 (22.4%)	13 (21.7%)	13 (23.2%)	0.842
*Dysarthria / slurred speech*	24 (20.7%)	10 (16.7%)	14 (25.0%)	0.271
*Blurred vision*	23 (19.8%)	9 (15.0%)	14 (25.0%)	0.181
*Ataxia/ gait abnormality*	21 (18.1%)	14 (23.3%)	7 (12.5%)	0.135
*Loss of memory*	18 (15.5%)	10 (16.7%)	8 (14.3%)	0.724
*Vertigo/ dizziness*	16 (13.8%)	8 (13.3%)	8 (14.3%)	0.882
*Neck stiffness*	15 (12.8%)	3 (5.0%)	12 (21.4%)	0.015
*Asphyxia*	14 (12.1%)	6 (10.0%)	8 (14.3%)	0.481
*Chest pain*	14 (12.1%)	12 (20.0%)	2 (3.6%)	0.016
*Forced gaze (conjugate deviation)*	12 (10.3%)	6 (10.0%)	6 (10.7%)	0.900
*Coma*	11 (9.5%)	2 (3.3%)	9 (16.1%)	0.033
*Altered sensorium*	9 (7.8%)	6 (10.0%)	3 (5.4%)	0.358
*Convulsion/ seizure /abnormal body movement*	8 (6.9%)	5 (8.3%)	3 (5.4%)	0.530
*Visual field defect*	7 (6.0%)	4 (6.7%)	3 (5.4%)	0.768
*Trismus (lock jaw)*	5 (4.3%)	2 (3.3%)	3 (5.4%)	0.228
*Diplopia*	5 (4.3%)	2 (3.3%)	3 (5.4%)	0.595
*Monoparesis/plegia*	2 (1.7%)	0 (0%)	2 (3.6%)	0.999

*OR* Odds ratio

Hemorrhagic stroke patients were more likely to be presented with coma (*P* = 0.033), vomiting (*P* = 0.028) and neck stiffness (*p* = 0.015), but ischemic stroke patients were more likely presented with chest pain (*p* = 0.016). In other clinical presentations there was no statistical significant difference between stroke subtypes. The average clinical presentation per patient was 6 (ranged from 2 to 12).

### Predictors of stroke subtypes

Using *P* < 0.05 for candidate variable selection for predictors of stroke subtypes on binary logistic regression; atrial fibrillation, heart failure, previous stroke, coronary disease, smoking, migraine/headache and previous situation of hypertension management were selected to be included in multivariable logistic regression. Up on multivariable logistic regression only atrial fibrillation (AOR: 0.08; 95% CI: 0.01–0.68, P: 0.021) was the independent predictor for hemorrhagic stroke. Patients having atrial fibrillation were 0.08 times less likely experience hemorrhagic stroke than ischemic stroke **(**Table 4**).**

**Table 4:** Predictors of hemorrhagic stroke compared to ischemic stroke patients admitted to stroke unit of JUMC

Variables		HS	IS	COR 95% CI	*P* value	AOR 95% CI	*P* value
Atrial fibrillation	*Yes*	4	15	0.231 (0.71–0.75)	0.014	0.08 (0.01–0.68	0.021*
	*No*	51	45	1.00		1.00	
Heart failure	*yes*	4	16	0.21 (0.07–0.68)	0.009		
	*no*	52	44	1.00			
Previous stroke	*yes*	1	8	0.12 (0.01–0.98)	0.048	0.18 (0.02–1.66)	0.130
	*no*	55	52	1.00		1.00	
Smoking (current/previous)	*yes*	14	6	3.00 (1.06–8.47)	0.038		
	*no*	52	54	1.00			
Coronary disease (CHD, IHD)	*yes*	3	11	0.25 (0.67–0.98)	0.043		
	*no*	53	49	1.00			
Migraine/ headache history	*yes*	10	2	6.30 (1.32–30.20)	0.021	14.54 (0.85–249.98)	0.065
	*no*	46	58	1.00		1.00	
situation of previous HTN management	*Medication discontinued*	15	9	3.33 (1.06–10.53)	0.04		
	*Not start medication*	10	9	2.22 (0.67–7.41)	0.194		
	*On medication*	9	18	1.00			

*AOR* Adjusted odds ratio, *CHD* Coronary Heart Disease, *COR* Crude odds ratio, *IHD* Ischemic Heart Disease, *HTN* Hypertension

*stastistically significant at *P* < 0.05

## Discussion

This study data was drawn from the huge study project done on stroke in SU of JUMC. The study populations participated in this finding share similarity with previously published articles of the same project [23, 24]. Even though this study shared similarity and textual overlap in the method and the socio-demographic part with previous findings, this finding provides advance and unique contribution over the previous published studies by exploring the risk factors and clinical presentation of stroke.

The mean age of the patients (55.1 ± 14.0 years), was in line with other studies carried out in developing countries including Ethiopia [29, 32–36], but lower compared to studies by Tirschwell et al. and Sagui et al. [37, 38]. In developing countries like Ethiopia, stroke occurs a few years earlier as compared to developed countries. This disagreement may be due to liability of hospital based studies to selection bias, demographic differences (differences in birth rates and survival into old age) and poor risk factor control. Thus community based studies are required to clearly find out and compare incidence as well as prevalence of stroke by age in our area. Young stroke (< 45 Years) comprised of more than one fifth (22.4%) of all patients similar to study in other part of Ethiopia [36], but higher than study in Gujarat, Nigeria and other parts of Ethiopia [21, 22, 32, 39].

The higher percentage of stroke in male patients over females was in line with other previous studies [14, 17, 29, 30, 39]. The possible reason may be increased risk factors such as cigarette smoking and alcohol consumption among males. In addition, there is no vascular protection of endogenous estrogens in males. This was unlike to some studies where female patients were dominant [13, 22]; may be due high use of contraception, pregnancy related disorders and migraine causing stroke among females in those studies. In our study finding majority of the patients were rural residents. Contrary to this, findings by Gebremariam et al. [21] and Greffie et al. [22] showed that majority of the patients were from urban areas. It is clear that hospital-based cohorts differ in the type of persons that come to the hospital. The location and catchment area of the hospital determines category of patients visiting the hospital. Additionally, cities and rural regions may differ in age constituencies. The high burden of stroke in rural population may also be due to reduced awareness and poor control of risk factors.

Majority of the patients were farmers (37.9%) and housewives (35.3%), which correlates with the study in Nigeria [40], but contrary to studies in Zambia and Vietnam [37, 41]. Lack of information, ignorance of the risk factors and inability to manage such risk factors might be responsible to this effect. Even when the patients understand the risk factors, they may not accept them to be the cause for stroke nor be able to afford the cost of medications. Additionally, since managing risk factors of stroke require longer period or may be life time; most patients failed to adhere and follow it properly. The above causes might have contributed in many directions to the high prevalence of stroke among peoples with lower educational level including housewives and farmers.

In this study majority of the patients (63.8%) had normal BMI and only 15.5% of the patients were overweight. Majority of the patients in developing countries had low or normal BMI because of low economic status and have increased labor related physical activities. Compared to normal weight patients, obese and overweight patients are susceptible to develop a stroke. This may be associated with increased risk factors, insulin resistance, pro-thrombotic state, excessive secretion of free fatty acids, release of excitatory amino acids and sympathetic nervous system activation. This directly or indirectly related to thrombotic and coagulation adverse events thereby reducing the functional outcome and may result in catabolic imbalance. At the same time, immobilization in obese patients can impair the post stroke recovery and outcome.

The most common risk factor identified was hypertension in 75.9%, consistent with other findings as uncontrolled hypertension is the most important risk factor for stroke both in developing and developed countries [12, 13, 29, 30, 32, 36, 37]. This trend may reflect poor community awareness, health practices and access to healthcare including different patient related factors. Even when blacks are treated for hypertension, they are less likely than white races to be adhere with treatments given for them. This leads us to believe that hypertension is underdiagnosed and less treated in our study community due to lack of an active screening program, failure to take routine blood pressure measurements, poor medical history taking and poor follow up of the patients. Additionally, adherence with long-term treatment is a great challenge to achieve the optimum outcome as uncomplicated hypertension is usually asymptomatic and denial of the disease is common.

In this study, 79.5% of the hypertensive patients had a pre-stroke knowledge of being hypertensive and 27(38.6%) were on anti-hypertensive medication prior to the stroke occurrence. This was in line with study by Gebremariam et al. in which 20 (37.0%) of the patients had prescribed anti-hypertensive medication prior to the stroke occurrence [21]. But it was in contrary to study by Watila et al. [32] in which more than half of the patients had no prior knowledge of being hypertensive and only small proportion of patients had treatment for hypertension prior to having a stroke. The median duration of hypertension prior to stroke was 3 years, in line with previous study in Ethiopia by Gebremariam et al. [21].

From patients who were on antihypertensive medication during hospital arrival, in majority of the patients’ blood pressure was not controlled (≥ 140/90 mmHg). Poor control of blood pressure is associated with adherence problem, lack of frequent monitoring, cost issue for medications and transportation for follow up. The proportion of patients that never had their blood pressure measured was lower than finding by Walker etal [33]. Most patients discontinued their antihypertensive medications by convincing themselves as they were cured or improved, because hypertension is asymptomatic disease until organ damage is evident.

Diabetes mellitus is one of the major risk factor for the development of atherosclerosis and the excess risk of stroke. It was diagnosed as co-morbidity in 8 patients, without statistically significant difference between stroke subtypes. According to study by Alemayehu et al. infarction is the most common type of stroke events in diabetic individuals (57.7%) [13]. In our study the prevalence of DM was lower compared to study by Sarkar et al. (25.9%) [34], 46.8% by De Carvalho etal [42], 23.8% by Desalu etal [43], 19.5% by Owolabi etal [17] and 10.1% by Watila etal [32]. But was closely similar with study by Deresse et al. in Ethiopia which was identified in 7.8% of stroke patients [29]. This discrepancy could be due to our small sample size, referral bias and single hospital-based design of our study. We recommend well designed multi-centered studies to quantify the risk of diabetes in Ethiopian stroke patients. The mean duration of diabetes prior to stroke was 5.3 year, that was closely correlates with study by Gebremariam etal [21].

Habituation of alcohol (22.4%) and smoking (17.2%) was higher compared to other previous studies [14, 17, 32, 39, 43]. This was mostly associated with the community in catchment area of our hospital were highly abuser of social drugs. The majority of smokers develop stroke due to smoking may predispose blood vessels to thrombosis and facilitates platelets aggregation possibly by causing an imbalance between brain vascular coagulation and abnormal fibrinolysis. This might alter the function of blood brain barrier and disrupt normal endothelial cell function. The relation between alcoholism and risk factor of stroke is more susceptible to aggravating effect which causes cardio embolism and hypertension thereby increases the risk of ischemic stroke.

In this study 12.9% of patients were previous user of diet containing low fruit and vegetable. The relation between risk of stroke and diet may be associated with increased daily total fat intake that greatly increases risk of stroke. But vegetable foods have low saturated fat and are protective for our health and organ function. Similar to previous study by Tirschwell et al. [37] cardiac disease like atrial fibrillation, coronary disease and heart failure were commonly associated with ischemic stroke than hemorrhagic strokes. Atrial fibrillation which is great source of cardioembolic stroke was diagnosed in 16.4% that was consistent with study by De Carvalho etal 14.95% [42] and Sagui etal 14.7% [38].

Up on multivariate logistic regression, atrial fibrillation was the independent predictor for hemorrhagic stroke. Patients having atrial fibrillation were less likely experience hemorrhagic stroke than ischemic stroke. From the pathophysiology of the stroke, atrial fibrillation is the most common reason for cardioembolic stroke that occludes cerebral arteries which favors ischemic stroke over hemorrhagic stroke. This finding complies with study by Atadzhanov etal in Zambia [16, 41].

At the onset of stroke, the most common clinical presentation was headache (75.0%) followed by aphasia (60.3%) and hemiparesis (53.4%), similar finding was reported on study by Walker et al. in Gambia [33]. This finding was unlike to other studies where motor symptoms (hemiplegia/hemiparesis) were the most common clinical presentation among stroke patients [13, 14, 22, 35, 39, 42]. The difference could be due to two major reasons. Primarily we have collected data on motor symptoms separately; hemiparesis and hemiplegia. Thus if we had collected as one category the result complies with those other previous studies, as 82.6% of patients manifest either hemiplegia/hemiparesis. Secondly even though the severity varies in degrees due to the nature of the disease most patients may complain the headache as the study was prospective with face to face interview. Initial presentation of urinary incontinence was higher (37.9%) as compared to other study by Greffie et al. [22]. Aphasia was one common presentation in this study which was less presentation as compared to other previous studies [14, 22, 39]. Similar to our finding, study by Kuriakose etal [7] reported that vomiting favors hemorrhagic stroke. This may be one indicator of stroke diagnosis based on clinical where brain imaging is not available. In general average clinical presentation for the patient was 6, which was higher than study in India by Kuriakose et al. in which majority of the patients had 3–4 clinical manifestation during admission [7].

### Strength and limitations of the study

This study attempted to identify different risk factors related to stroke with a prospective clinical follow-up that focused on the need of preventive strategy and improvement of patient care. To ensure a uniform data collection, we ascertained consistently ascertainable risk factor identification and obtained more or less reliable information to achieve the goals of our study. More generable case ascertainment than in earlier studies, in-person health care professional assessment to verify eligibility for inclusion was addressed.

The study was associated with some limitations and drawbacks. First, this study was a hospital-based study rather than longitudinal community based study. Hence it may be subjected to referral bias, as most of the acute stroke patients’ visit our hospital only from the south western part of Ethiopia. These referral bias as well as convenience sampling approach used might not reflect the true prevalence of the stroke in the community. Even though the study was hospital based, having only one referral center might probably reflect the actual magnitude of stroke in our country.

Secondly, about half of the patients were diagnosed clinically alone to have stroke based on clinical presentations, risk profiles, disease course and other supportive investigations. Clinical way of diagnosis based on clinician judgment rather than biological may distort accuracy and reliability of the data. This may cause unintended false positive and false negative association between different variables of the study. Thus caution should be taken for the generalization of the finding for large community.

Thirdly, in our study protocol, the risk factor status was not refined sufficiently enough especially for ischemic stroke patients with cardiac cases. Even simple and inexpensive diagnostic tests like electrocardiograms (ECG) were not routinely performed. Poor risk factor identification and diagnosis may underestimate or overestimates some factors. Finally, the sample size was small hampering the analysis of some prognostic indicators due to the short recruitment period. In addition, we counted on patient reports of some of their risk factors and other patient related histories, which may introduce recall bias.

## Conclusion

Majority of the patients were males, middle aged, rural residents, uneducated and farmers with low socioeconomic status. The increasing burden of stroke in LMICs countries like Ethiopia poses a challenge to the health care system and the community as a whole. The most common risk factor identified was hypertension and the level of poor blood pressure control in hypertensive patients we observed in this study was alarming. The most common clinical presentation was headache and motor symptoms (hemiplegia/hemiparesis). Hemorrhagic stroke patients were more likely to have coma, vomiting and neck stiffness but ischemic stroke patients were more likely presented with chest pain.

As stroke is a high priority chronic global case, large-scale community health campaign should be launched focusing on community education regarding risk factors of stroke as well as recognition of stroke-related symptoms, prognosis and outcomes. The importance of early recognition and treatment may help to improve outcomes, facilitate consistent and continuous follow up as well as with available treatment options disability can be minimized. Educational programs for front-line health-care providers, focusing on simple supportive interventions, could improve outcomes in settings where advanced diagnostics and treatment of stroke remain limited.

In addition, there should be influential contribution from every social media and political level of the country with the goal of increasing the awareness of risk factors and making the community to understand the challenging effect of the stroke on human health and economy of the country. Thus, policy makers should put strategies for screening and management of common risk factors like hypertension.

## Additional file

Additional file 1: Data abstraction tool. (DOCX 24 kb)

## Data Availability

The datasets used and/or analyzed during the current study are available from the corresponding author on reasonable request.

## Abbreviations

AF: Atrial fibrillation
BMI: Body Mass Index
DM: Diabetes Mellitus
GBD: Global Burden of Diseases
HS: Hemorrhagic stroke
IHD: Ischemic heart Disease
IS: Ischemic stroke
JUMC: Jimma university medical center
LMICs: Low and middle income countries
SSA: Sub Saharan Africa
SU: Stroke unit
WHO: World Health Organization

## References

[1] Investigators WMPP (1988). The world health organization monica project (monitoring trends and determinants in cardiovascular disease): a major international collaboration. J Clin Epidemiol.

[2] National Collaborating Centre for Chronic Conditions (UK) (2008). Stroke: National Clinical Guideline for Diagnosis and Initial Management of Acute Stroke and Transient Ischaemic Attack (TIA).

[3] Jowi JO, Mativo PM (2008). Pathological sub-types, risk factors and outcome of stroke at the Nairobi hospital, Kenya. East Afr Med J.

[4] Ekeh B, Ogunniyi A, Isamade E, Ekrikpo U (2015). Stroke mortality and its predictors in a Nigerian teaching hospital. Afr Health Sci.

[5] Feigin VL, Forouzanfar MH, Krishnamurthi R, Mensah GA, Connor M, Bennett DA, Moran AE, Sacco RL, Anderson L, Truelsen T (2014). Global and regional burden of stroke during 1990-2010: findings from the global burden of disease study 2010. Lancet (London, England).

[6] Mozaffarian D, Benjamin EJ, Go AS, Arnett DK, Blaha MJ, Cushman M, Das SR, de Ferranti S, Despres JP, Fullerton HJ (2016). Heart disease and stroke Statistics-2016 update: a report from the American Heart Association. Circulation.

[7] Kuriakose C, Shifafiya MN, Tharakan NS, K S, Kumar RS (2016). A prospective study of clinical profile of stroke in a tertiary care hospital. Asian J Pharm Clin Res.

[8] Feigin VL, Norrving B, Mensah GA (2017). Global burden of stroke. Circ Res.

[9] Feigin VL, Krishnamurthi RV, Parmar P, Norrving B, Mensah GA, Bennett DA, Barker-Collo S, Moran AE, Sacco RL, Truelsen T (2015). Update on the global burden of ischemic and hemorrhagic stroke in 1990-2013: the GBD 2013 study. Neuroepidemiology.

[10] Moran A, Forouzanfar M, Sampson U, Chugh S, Feigin V, Mensah G (2013). The epidemiology of cardiovascular diseases in sub-Saharan Africa: the global burden of diseases, injuries and risk factors 2010 study. Prog Cardiovasc Dis.

[11] Boehme AK, Esenwa C, Elkind MSV (2017). Stroke risk factors, genetics, and prevention. Circ Res.

[12] Zewdie A, Debebe F, Kebede S, Azazh A, Laytin A, Pashmforoosh G, Hassen GW (2018). Prospective assessment of patients with stroke in Tikur Anbessa specialised hospital, Addis Ababa, Ethiopia. African J Emerg Med.

[13] Alemayehu CM, Birhanesilasie SK (2013). Assessment of stoke patients: occurrence of unusually high number of haemorrhagic stroke cases in Tikur Anbessa specialized hospital, Addis Ababa, Ethiopia. Clin Med Res.

[14] Patne SV, Chintale KN (2016). Study of clinical profile of stroke patients in rural tertiary health care Centre. Int J Adv Med.

[15] Owolabi MO, Sarfo F, Akinyemi R, Gebregziabher M, Akpa O, Akpalu A, Wahab K, Obiako R, Owolabi L, Ovbiagele B (2018). Dominant modifiable risk factors for stroke in Ghana and Nigeria (SIREN): a case-control study. Lancet Glob Health.

[16] Namale G, Kamacooko O, Kinengyere A, Yperzeele L, Cras P, Ddumba E, Seeley J, Newton R (2018). Risk factors for hemorrhagic and ischemic stroke in sub-Saharan Africa. J Trop Med.

[17] Owolabi MO, Agunloye AM (2013). Which risk factors are more associated with ischemic rather than hemorrhagic stroke in black Africans?. Clin Neurol Neurosurg.

[18] Owolabi MO, Akarolo-Anthony S, Akinyemi R, Arnett D, Gebregziabher M, Jenkins C, Tiwari H, Arulogun O, Akpalu A, Sarfo FS (2015). The burden of stroke in Africa: a glance at the present and a glimpse into the future. Cardiovasc J Afr.

[19] Al-Hashel JY, Al-Sabah AA, Ahmed SF, Al-Enezi M, Al-Tawheid N, Al Mesailekh Z, Eliwa J, Alroughani R (2016). Risk factors, subtypes, and outcome of ischemic stroke in Kuwait: a National Study. J Stroke Cerebrovasc Dis.

[20] Abate AT, Bayu N, Mariam TG (2019). Hypertensive Patients’ Knowledge of Risk Factors and Warning Signs of Stroke at Felege Hiwot Referral Hospital, Northwest Ethiopia: A Cross-Sectional Study. Neurol Res Int.

[21] Gebremariam SA, Yang HS (2016). Types, risk profiles, and outcomes of stroke patients in a tertiary teaching hospital in northern Ethiopia. eNeurologicalSci.

[22] Greffie ES, Mitiku T, Getahun S (2015). Risk factors, clinical pattern and outcome of stroke in a referral hospital, Northwest Ethiopia. Clin Med Res.

[23] Fekadu G, Wakassa H, Tekle F (2019). Stroke Event Factors among Adult Patients Admitted to Stroke Unit of Jimma University Medical Center: Prospective Observational Study. Stroke Res Treat.

[24] Fekadu G, Chelkeba L, Melaku T, Tegene E (2018). Pathological sub types and diagnostic protocols of stroke among adult patients admitted to Jimma University medical center, south West Ethiopia. J Neurol Neurophysiol.

[25] STEPS-Stroke. The WHO step wise approach to stroke surveillance. Geneva: 2006 [cited 2017 March 28]; Available from: http://www.who.int/ncd_surveillance/en/steps_stroke_manual_v1.2.pdf.

[26] Wu CY, Wu HM, Lee JD, Weng HH (2010). Stroke risk factors and subtypes in different age groups: a hospital-based study. Neurol India.

[27] Jia XY, Huang M, Zou YF, Tang JW, Chen D, Yang GM, Lu CH (2016). Predictors of poor outcomes in first-event ischemic stroke as assessed by magnetic resonance imaging. Clin Invest Med.

[28] Manorenj S, Inturi S, Jyotsna B, Sai Savya V, Areli D, Balarami Reddy O (2016). Prevalence, pattern, risk factors and outcome of stroke in women: a clinical study of 100 cases from a tertiary care center in South India. Int J Res Med Sci.

[29] Deresse B, Shaweno D (2015). Epidemiology and in-hospital outcome of stroke in South Ethiopia. J Neurol Sci.

[30] Nkoke C, Lekoubou A, Balti E, Kengne AP (2015). Stroke mortality and its determinants in a resource-limited setting: a prospective cohort study in Yaounde, Cameroon. J Neurol Sci.

[31] Gezmu T, Schneider D, Demissie K, Lin Y, Gizzi MS (2014). Risk factors for acute stroke among south Asians compared to other racial/ethnic groups. PLoS One.

[32] Watila MM, Nyandaiti YW, Ibrahim A, Balarabe SA, Gezawa I, Bakki B, Tahir A, Sulaiman MM, Bwala SA (2012). Risk factor profile among black stroke patients in northeastern Nigeria. Neurosci Biobehav Rev.

[33] Walker RW, Rolfe M, Kelly PJ, George MO, James OF (2003). Mortality and recovery after stroke in the Gambia. Stroke.

[34] Sarkar D, Halder S, Saha BK, Biswas P (2016). A study of stroke patients with respect to their clinical and demographic profile and outcome. Int J Res Med Sci.

[35] Masood CT, Hussain M, Anis ur R, Abbasi S (2013). Clinical presentation, risk factors and outcome of stroke at a district level teaching hospital. J Ayub Med Coll Abbottabad.

[36] Bekele A, Kebede O (2002). Stroke admission to Tikur Anbessa teaching hospital: with emphasis on stroke in the young. Ethiop J Health Dev.

[37] Tirschwell DL, Ton TG, Ly KA, Van Ngo Q, Vo TT, Pham CH, Longstreth WT, Fitzpatrick AL (2012). A prospective cohort study of stroke characteristics, care, and mortality in a hospital stroke registry in Vietnam. BMC Neurol.

[38] Sagui E, M'Baye PS, Dubecq C, Ba Fall K, Niang A, Gning S, Bellefleur JP, Sane M, Debonne JM (2005). Ischemic and hemorrhagic strokes in Dakar, Senegal: a hospital-based study. Stroke.

[39] Vaidya CV, Majmudar DK (2014). A retrospective study of clinical profile of stroke patients from GMERS medical college and hospital, Gandhinagar, Gujarat. Int J Clin Trials.

[40] Njoku CH, Aduloju AB (2004). Stroke in Sokoto, Nigeria: a five year retrospective study. Ann Afr Med.

[41] Atadzhanov M, Mukomena P, ShabirLakhi ROA, Meschia JF (2012). Stroke characteristics and outcomes of adult patients admitted to the university teaching hospital, Lusaka, Zambia. Open Gen Intern Med J.

[42] De Carvalho JJ, Alves MB, Viana GA, Machado CB, dos Santos BF, Kanamura AH, Lottenberg CL, Neto MC, Silva GS (2011). Stroke epidemiology, patterns of management, and outcomes in Fortaleza, Brazil: a hospital-based multicenter prospective study. Stroke.

[43] Desalu OO, Wahab KW, Fawale B, Busari OA, Adekoya AO, Afolayan JO, Olarenwaju TO (2011). A review of stroke admissions at a tertiary hospital in rural southwestern Nigeria. Ann Afr Med.

