# Supplementary material for: Risk factors, clinical presentations and predictors of stroke among adult patients admitted to stroke unit of Jimma university medical center, south west Ethiopia: prospective observational study
Source: BMC Neurol. 2019 Aug 7;19:187. doi: 10.1186/s12883-019-1409-0 (PMC6685251; doi:10.1186/s12883-019-1409-0)
Supplement: Supplementary file 1 — Data abstraction tool. (DOCX 24 kb) [file 12883_2019_1409_MOESM1_ESM.docx]

**Additional file 1: Data abstraction tool**

| **Abstraction done by: _________________________________________________________date*___________________*** | | | | | | | | | | | | | | |  |
| --- | --- | --- | --- | --- | --- | --- | --- | --- | --- | --- | --- | --- | --- | --- | --- |
| **Source of history for the interview:** Solely from the patients  From the patient and caregiver or other informant  Solely from relatives and/or friends/care giver | | | | | | | | | | | | | | |  |
| 1. **Patient socio-demographic information** | | | | | | | | | | | | | | |  |
| MRN/card number :__________ | Date of admission: ______________ | | **Age**:______ (yrs) | | | **Sex:**   - Male - Female | | | Kebele:__________  Woreda:_________  Region:________ | | | | | Residence   - - Urban   - rural |  |
| **Marital status**   - single - Married - Divorced - Widow | **Ethnicity**   - Oromo - Amhara - Kefa - Other____________ | | **Education status**   - Unable to read and write - Able to read and write, informal education (eg religious education) (Less than primary school) - Elementary school (1-8) - Secondary school (9-12) - College/university or above | | | | | | **Religion**   - Orthodox - Protestant - Muslim - Catholic - Traditional belief - Others_________ | | | | | - Weight(kg)________ - Height (cm):_______ - BMI:____________ |  |
| **Occupational status (**over the last 1years)   - Government employee - Non-government employee - Merchant - Other own business work - Skilled/unskilled manual labor/ daily worker - Agriculture / farmer - Non-paid (eg.volunteer) - Student - Homemaker/ housewives - Retired - Unemployed - Other(specify)______________________ | | |  |  |  |  |  |  |  |  |  |  |  |  |  |
|  |  |  |  |  |  |  |  |  | **Living situation of patient during pre-stroke**   - Independent at home - Dependent at home - Community facility - hospital/ health center - Other:______________ | | | | | |  |
|  |  |  | **Food habits**  ☐ Vegetarian  ☐ Non vegetarian  ☐ Mixed diet | | | | | |  |  |  |  |  |  |  |
| 1. **Risk factors/ causes** | | | | | | | | | | | | | | |  |
| - Hypertension   - Newly diagnosed - No history of hypertension   - *had never had their blood pressure measured*   - *measured but normal range* - have history of hypertension/ awareness of hypertension   - *duration when they know as they have HTN_______*   - *if taking medication, duration of starting anti-hypertensive_____________*   - *Not taking any medication/or discontinued*   *If currently on antihypertensive: on admission*   - - *BP not controlled (>140/90 on >2 measurements)*   - *BP controlled(<140/90)*   NB: control diabetes can be RBS > 200 mg/dl and hyperlipidemia can be by total cholesterol > 200mg/dl, serum triglycerides > 150 mg/dl | | | | | - Diabetes mellitus: (duration)_______   If on antidiabetic: on admission   - *BG controlled (FBS 70-130 mg/dl* - *BG not controlled ( FBS>130 mg/dl)* | | | | | | - alcohol intake / abuse   - *never*   - *former drinker before one year*   - *current alcohol use* | | | |  |
|  |  |  |  |  | - Hyperlipidemia / duration)________   If on lipid lowering agent: at admission   - *lipid controlled ( LDL<100mg/dl)* - *Lipid not controlled ( LDL >100 mg/dl)* | | | | | | - physical activity - *Work related aerobic physical activity* - *Aerobic/planned physical activity* - *No physical activity* | | | |  |
|  |  |  |  |  | - Overweight/Obesity (based on BMI and waist circumference) | | | | | |  |  |  |  |  |
|  |  |  |  |  |  |  |  |  |  |  | - Drug abuse (e.g., cocaine, amphetamine, etc) | | | |  |
|  |  |  |  |  | - Smoking / Tobacco use - *current smoker* - *former smoker before one year* - *never smoked* | | | | | |  |  |  |  |  |
|  |  |  |  |  |  |  |  |  |  |  | - Diet (low fruit and vegetable) | | | |  |
| - Contraceptive drug use | - Family history of   - *Sudden death*   - *Stroke*   - *Ischemic heart disease*   - *DM*   - *HTN*   - *Other___________* | | - Previous stroke/TIA | | | | | | - PAD | | | | - Hematological disorders - *Hematological Malignancy* - *Polycythaemia* - *Thromobocytosis* - *Thrombocytopenia* - *Anemia* | |  |
| - AF or flutter |  |  | - Hypertensive heart disease (HHD) | | | | - Sickle cell disease | | - Chronic liver disease | | | |  |  |  |
| - heart failure |  |  |  |  |  |  |  |  |  |  |  |  |  |  |  |
| - Valvular heart disease (RHD, prosthetic heart valve, Mechanical heart valve) |  |  | - Coronary disease (CHD, IHD) | | | | - Hyperuricemia | | - Oral anticoagulants | | | |  |  |  |
|  | - Epilepsy/seizure | |  |  |  |  |  |  |  |  |  |  | - Other Malignancy/ H/o of other cancer (_______) | |  |
|  | - Infective meningitis (tuberculosis meningitis or bacterial meningitis) | | - Other cardiovascular diseases such as DCM | | | | | | - VTE/hypercoagulable sates | | | |  |  |  |
|  |  |  |  |  |  |  |  |  |  |  |  |  | - Thyrotoxicosis | |  |
|  |  |  | - Psychosocial stress | | | | | | - Traumatic brain /head injury | | | |  |  |  |
| - Nephrotic syndrome/Polycystic kidney disease | | | - COPD | | | | - SLE | |  |  |  |  |  |  |  |
|  |  |  |  |  |  |  |  |  | - carotid stenosis | | | | - Miscarriage | |  |
| - preeclampsia-eclampsia or other pregnancy related disorders | | | - Migraine or headache | | | | - Syphilis | | - HIV infection | | | |  | |  |
|  |  |  |  |  |  |  |  |  | - Other comorbidities | | | | - No identified risk factors | |  |
|  |  |  | - Other_________________________ | | | | | | | Number of risk factor____________ | | | | |  |
| 1. **Clinical presentation** | | | | | | | | | | | | | | |  |
| - Hemiparesis | | - Headache | | | | | | - Decreased level of consciousness | | | | - loss of memory | | | |
| - Hemiplegia | | - trismus (lock jaw) | | | | | | - Asphyxia | | | | - neck stiffness | | |  |
| - Paraplegia | | - Vertigo/ dizziness | | | | | | - Vomiting | | | | - Coma | | |  |
| - Quadriparesis/ general weakness | | - Visual field defect | | | | | | - Chest pain | | | | - Facial palsy | | |  |
| - Quadriplegia/ general paralysis | | - Swallowing difficulty / dysphagia | | | | | | - Dysarthria / slurred speech | | | | - Convulsion/ seizure/ABM | | |  |
| - Monoparesis/plegia | | - Bladder/ urinary incontinence | | | | | | - Forced gaze (conjugate deviation) | | | | - Ataxia/gait abnormality | | |  |
| - Aphasia/ dysphasia | | - Blurred vision | | | | | |  |  |  |  | - No symptoms | | |  |
| - Diplopia | | - Altered sensorium/ numbness/ loss   of sensation | | | | | | - other specify___________________________________   ________________________________________ | | | | | | |  |
| 1. **Approaches of stroke diagnosis and stroke subtypes** | | | | | | | | | | | |  |  |  |  |
| **Was brain imaging done for stroke confirmation (CT Scan/ MRI)**   - No imaging done (only clinical diagnosis) - Yes: | | | | **Stroke diagnosis**   - - Ischemic stroke   - Hemorrhagic stroke (subarachnoid)   - Hemorrhagic stroke (intracerebral)   - Other stroke______________ | | | | | | | |  |  |  |  |
